# KDM2B regulates choline kinase expression and neuronal differentiation of neuroblastoma cells

**DOI:** 10.1371/journal.pone.0210207

**Published:** 2019-01-10

**Authors:** Pablo Domizi, Florencia Malizia, Lorena Chazarreta-Cifre, Lautaro Diacovich, Claudia Banchio

**Affiliations:** Instituto de Biología Molecular y Celular de Rosario (IBR, CONICET) Ocampo y Esmeralda, Predio CONICET and Facultad de Ciencias Bioquímicas y Farmacéuticas, Universidad Nacional de Rosario, Rosario, Argentina; University of South Alabama Mitchell Cancer Institute, UNITED STATES

## Abstract

The process of neuronal differentiation is associated with neurite elongation and membrane biogenesis, and phosphatidylcholine (PtdCho) is the major membrane phospholipid in mammalian cells. During neuroblast differentiation, the transcription of two genes involved in PtdCho biosynthesis are stimulated: *Chka* gene for choline kinase (CK) alpha isoform and *Pcyt1a* gene for CTP:phosphocholine cytidylyltransferase (CCT) alpha isoform. Here we show that CKα is essential for neuronal differentiation. In addition, we demonstrated that KDM2B regulates CKα expression and, as a consequence, neuronal differentiation. This factor is up-regulated in the course of the neuroblasts proliferative and undifferentiated state and down-regulated during differentiation induced by retinoic acid (RA). During proliferation, KDM2B binds to the Box2 located in the *Chka* promoter repressing its transcription. Interestingly, KDM2B knockdown enhances the levels of CKα expression in neuroblast cells and induces neuronal differentiation even in the absence of RA. These results suggest that KDM2B is required for the appropriate regulation of CKα during neuronal differentiation and to the maintaining of the undifferentiated stage of neuroblast cells.

## Introduction

In mammalian cells, the supply of PtdCho can be regulated by biochemical activity of key enzymes [1,2], gene expression of biosynthetic or degradative enzymes [2,3,4,5,6] or intracellular trafficking [7,8]. It was previously demonstrated that during RA-induced differentiation of Neuro-2a cells, the increase in PtdCho biosynthesis is promoted by an ordered and coordinated stimulation of CKα and CCTα activity and expression. Particularly, CKα expression is low in neuroblasts, and its transcription initiates 24 h after retinoic acid (RA) induction, leading to an increase in CKα levels [9]. The transcription of *Chka* gene during neuronal differentiation induced by RA depends on ERK_1/2_ activation and on the binding of C/EBPβ to the Box1 in the *Chka* promoter [10]. It was also demonstrated that enforced expression of CKα increases the rate of synthesis and the amount of PtdCho, initiating the differentiation of these cells, without RA stimulation [9].

Epigenetic regulation is pivotal in cell lineage decisions. These events, including DNA methylation and posttranslational covalent modifications of histones that modifies chromatin structure, selectively activate or repress the transcription of a subsets of tissue-specific genes. Polycomb group proteins are conserved chromatin proteins that contribute to gene silencing throughout higher eukaryotes [11,12]. KDM2B (also called FBXL10 or JHDM1B), originally known as a demethylase against the dimethylation at lysine 4 and 36 of histone H3 (H3K36me2), is a member of non-canonical polycomb repressor complex 1 (PRC1). This complex also regulates the gene expression by interacting with the RING-E3 ubiquitin ligase [13]. It was demonstrated that KDM2B plays critical roles in tumorigenesis and self-renewal of cancer stem cells in hematopoietic malignancies [14], in gynecological (breast, cervival and ovarian) cancers [15], and in pancreatic and gastric cancers [16]. It was also demonstrated that KDM2B regulates adipocyte differentiation [17] and prevents mouse embryonic stem cell differentiation [18].

Neuroblastoma is the most common extra-cranial malignant tumor of childhood [19], and is characterised by a wide heterogeneity in clinical presentation and evolution. In fact, tumors can spontaneously regress to a differentiated phenotype, or display an aggressive and therapy-resistant phenotype. These tumors are characterized by the failure of neuronal crest precursor cells to differentiate, which is the seed for neuroblast tumor formation. This tumor can sometime be treated with compounds that induce differentiation like retinoids (RA) and derivatives of vitamine A [20], however, there are many tumors resistant to RA-induced differentiation and these patients are not benefit with these therapies. The role of H3K27 demethylase in neuroblastoma was recently demonstrated [21] by showing that inhibition of its activity has profound effect on key differentiation genes and pathways associate with this tumor.

In this work, we demonstrated that CKα expression is essential for neuroblast cells differentiation. Moreover, we showed for the first time that KDM2B represses *Chka* expression through its binding to the inverted repeat region Box2 in the *Chka* promoter. High level of KDM2B and its mediated repression of CKα expression, are necessaries for keeping undifferentiated and proliferative neuroblast state. All together provides new information about the mechanism of *Chka*-transcriptional regulation during neuronal differentiation and the role of KDM2B in neuroblast cells.

## Material and methods

### Cell culture and treatments

The mouse neuroblastoma cell line Neuro-2a (ATCC CCL-131) was cultured in modified Eagle's medium (MEM), 10% fetal bovine serum (FBS) supplemented with penicillin G (100 units/ml), streptomycin (100 μg/ml) (proliferation conditions) and maintained in a 5% CO_2_ humidified incubator at 37°C. For neuronal differentiation, the medium was changed to Dulbecco's modified Eagle's medium (DMEM) plus 2% FBS and RA (10 μM). The human neuroblastoma cell line SH-SY5Y (kindly provided by Dr. S. Quiroga U. Of Córdoba) was cultured in a 1:1 mixture of DMEM and F12 medium, 10% fetal bovine serum (FBS) supplemented with penicillin G (100 units/ml), streptomycin (100 μg/ml), and maintained in a 5% CO2 humidified incubator at 37°C. In neuronal differentiation conditions, the amount of serum was changed to 1% FBS and supplemented with RA (10 μM).

Transient transfections with 5´ deletion of *Chka* promoter-luciferase reporter plasmids (0.5μg): Luc.CK(-1625/+57), Luc.CK(-901/+57), Luc.CKΔBox2 and Luc.CKΔBox1 were performed using a cationic liposome method (Invitrogen) [22]. All dishes received 0.2 μg of pCMV-β-galactosidase (Promega) as a control for transfection efficiency. Luciferase and β-galactosidase assays were performed using the Promega assay systems, as recommended by the manufacturer and luminometric measurements were made using Fluskan Ascent FL Type 374 (Thermolabsystems). Luciferase activity was normalized to β-galactosidase activity and expressed as a ratio Luciferase/ β-galactosidase.

### CKα and KDM2B knockdown

We designed two specific shRNAs targeting KDM2B or CK**α**, and a control shRNA (scrambled) flanked with BglII and HindIII enzyme sites (Table 1). The sequence of sh1-KDM2B was previously designed by Farcas et al. [23]. Self-complementary inverted repeat sequences were synthesized as single strand oligonucleotides by Invitrogen (Carlsbad, CA, USA), and then annealed and cloned into pSUPER vectors (pSUPER RNAi System™; OligoEngine Inc., Seattle, WA, USA), following manufacturer’s suggestions.

**Table 1:** 

Symbol	Characteristics	Specific sequence
sh1-CKα	shRNA designed for the knockdown of CKα	TACTTGACTACATTCCAAA
sh2-CKα	shRNA designed for the knockdown of CKα	ATTTGGGTACATGGAATAT
sh1-KDM2B	shRNA designed for the knockdown of KDM2B	CCCTGTGGAAATATCTGTCAT
sh2-KDM2B	shRNA designed for the knockdown of KDM2B	CCGCTCCAACTCAGTTACTGT

For transient transfections, cells were seeded in 6-well plates at a density of 50,000 cells/well. On the following day, cells were transfected with the plasmids using Lipofectamine 2000 (Thermo Fisher Scientific), according to the manufacturer’s instructions. After 48 h, pictures of the cells were taken for morphometric analysis and total cell homogenates were prepared for western blot.

### Isolation and identification of proteins bind to Box2

Nuclear extract from 3–4 x 10^7^ Neuro-2a cells grown under proliferation conditions was prepared as previously described [10]. Nuclear extracts were analyzed by size exclusion chromatography using an AKTÄ basic high-performance liquid chromatograph (GE) and a Superdex S200 column (GE). The column was equilibrated in 1X EMSA binding buffer (10 mM HEPES pH 7.9, 50 mM KCl, 2.5 mM MgCl_2_, 1 mM DTT, 1 mg/ml BSA, 10% glycerol) as running buffer. Samples containing nuclear extracts were loaded onto the columns and protein elutions, splited into 32 fractions, were followed by absorbance measurement at 220 nm. To identify those fractions that were able to bind to Box2 sequence, 20 μl from each fraction was incubated with 0.05 pmol P^32^ labeled Box2 probe and resolved by EMSA.

To purify Box2-binding proteins we used SoftLink Soft Release Avidin Resin (Promega) following manufacturer instructions. Those fractions, from size exclusion chromatography, that retained Box2 capacity were pooled together and incubated with 100 pmol of 5’ Biotinylated Box2 probe (GBT Oligos) 30 min at RT. Then, avidin resin was added to this mixture and incubated for further 30 min at RT. Resin was pooled down by centrifugation, washed three times with 1X EMSA binding buffer, and finally the complex biotinylated Box2-bound proteins were eluted by incubation with 5 mM free biotin in 1X EMSA binding buffer during 1 h at RT. The eluted proteins were analyzed by nLC-ESI-MS/MS spectrometer, in the mass spectrometry facility of the Structural Biology and Proteomic Service of Autonomous University of Barcelona.

### Morphometric analysis

Neuro-2a cells were plated at density of 3 x 10^4^ /35-mm dish for 24 h, after which the medium was changed to DMEM supplemented with 2% FBS and supplemented with RA (10 μM). Control cells were maintained in MEM 10% FBS. After 24 h, cells were observed by phase contrast microscopy (Olympus CK2) and 15–20 random fields of view were sampled. Cells bearing at least one neurite equal or longer than the soma diameter were considered to be differentiated. To calculate the percent of cells bearing neurites, differentiated cell and total cell number were counted and/or measured in each field, using the “ImageJ” (NIH) software. The concentration of the inhibitor was selected by MTT analysis [24]. When addition of inhibitors was necessary, it was added 60 min prior to the treatment.

### Western blot and immunofluorescence analysis

For western blot analysis, Neuro-2a cells were plated at a density of 5 x 10^5^ /100-mm dish for 24 h, after which the medium was changed to DMEM supplemented with 2% FBS and RA (10 μM). Control cells were maintained in MEM 10% FBS. After 48 h of treatment, cells were collected, resuspended in 1X lysis buffer (50 mM Tris-HCl pH 8.0, 50 mM KCl, 10 mM EDTA, 20 mM NaF, 1 mM Na_3_VO_4_, 1mM PMSF, 50 mM TPCK and 1:1000 cocktail (Sigma)) and sonicated five times for 5 s at 5% amplitude (Sonics and Materials Inc–Vibra Cell^TM^). In the case of SH-SY5Y cells, they were plated at a density of 2 x 10^5^ /100-mm dish in DMEM/F12 supplemented with 10% FBS. After 24 h the medium was changed to DMEM/F12 supplemented with 1% FBS and RA (10 μM). Control cells were maintained in DMEM/F12 10% FBS. After 4 and 6 days of treatment, cells were collected and resuspended in 1X lysis buffer previously described. Proteins concentrations were determined using bovine serum albumine (BSA) as standard protein and “Sedmak and Grossberg” reagent [25]. 20–40 μg of cell lysates were resolved on 7.5% SDS-polyacrilamide gel electrophoresis (PAGE) and transferred to a nitrocellulose membrane (Amersham). After blocking with 5% non-fat milk in 0.1% Tween TBS and washing, blots were incubated ON with anti-CKα (Abcam), anti-Flag (Sigma) or anti-KDM2B (Millipore). As secondary antibody was used peroxidise-conjugated anti-rabbit IgG (1:20000, Jackson Immuno Research). Loading protein control was demonstrated by measuring the levels of βtubulin using anti-βtubulin (1:1000, Sigma) or glyceraldehyde 3-phosphate dehydrogenase (GAPDH) (1:1000, Santa Cruz Biotechnology) and developed with secondary antibody peroxidase-conjugated anti-mouse IgG (1:10000, Jackson Immuno Research).

### Electromobility-shift assay (EMSA)

A probe of 25 pb (Invitrogen) were used for EMSA assays, and was designed over -920/-886 region of *Chka* promoter. Complementary oligonucleotides (10 μM of each) were heated at 90°C for 5 min and then slowly cooled at room temperature. 2.5 pmols of double stranded DNA probes were 5’-labeled using [γ^32^-P]-ATP and T4 polynucleotide kinase (Fermentas). For each binding reaction (20 μl): 4 μl of 5X binding buffer (50 mM HEPES pH 7.9, 250 mM KCl, 12,5 mM MgCl2, 5 mM DTT, 5 mg/ml BSA, 50% glycerol and 1 μg of poly-dI-dC), 5 μg of nuclear extract and labeled probe (50000 cpm) were incubated for 30 min at room temperature. Binding reactions were terminated by the addition of gel loading buffer (30% v/v glycerol, 0.1% w/v bromophenol blue, 0.1% w/v xylene cyanol). The complexes were separated on a non-denaturing 6% (w/v) polyacrylamide gel and visualized by autoradiography of the dried gel.

### Chromatin immunoprecipitation (ChIP) assay

Neuro-2a were grown in MEM containing 10% FBS for 24 h and incubated with 1% formaldehyde for 10 min at 37°C. Cells were collected, lysed, and sonicated during 10 min at medium intensity in the ultrasonic processor GEX-600 (Sonics & Materials) and treated for ChIP as recommended by the manufacturer (Upstate). We used a rabbit anti-KDM2B (Millipore) and the unrelated rabbit anti-βIII tubulin (Sigma) antibodies. For PCR analysis, we used ChIPCK forward primer (5´-AGTTTTTGGCTTCCAGCAGA-3´) and ChIPCK reverse primer (5´-ACATTAGTCATGGTCACGCG-3´) [10]. PCR was performed using 5 μl of template DNA, 1.5 mM MgCl_2_, and 20 pmol of each primer for 35 cycles at 94°C for 30 seg, 60°C for 30 seg, and 72°C for 30 seg.

### Kaplan curve

The Kaplan–Meier estimator for event free survival probability and overall survival was done using available online software R2: Kaplan Meier Scanner (https://hgserver1.amc.nl/cgi-bin/r2/main.cgi) and dataset from Kocak H, et al. Cell Death Dis 2013 (GSE45547). A total of 476 patients samples were separated between higher (17) and lower (459) KDM2B expression by scan mode in the cutoff_modus option.

### Statistical analysis

The data were analyzed using GraphPad Prism software. Significant effects were determined using Student’s t test, one or two–way ANOVA. A statistically significant difference was considered to be present when p < 0.05.

## Results

### Inhibition of choline kinase activity or expression impairs neuronal differentiation

**Fig 1:**
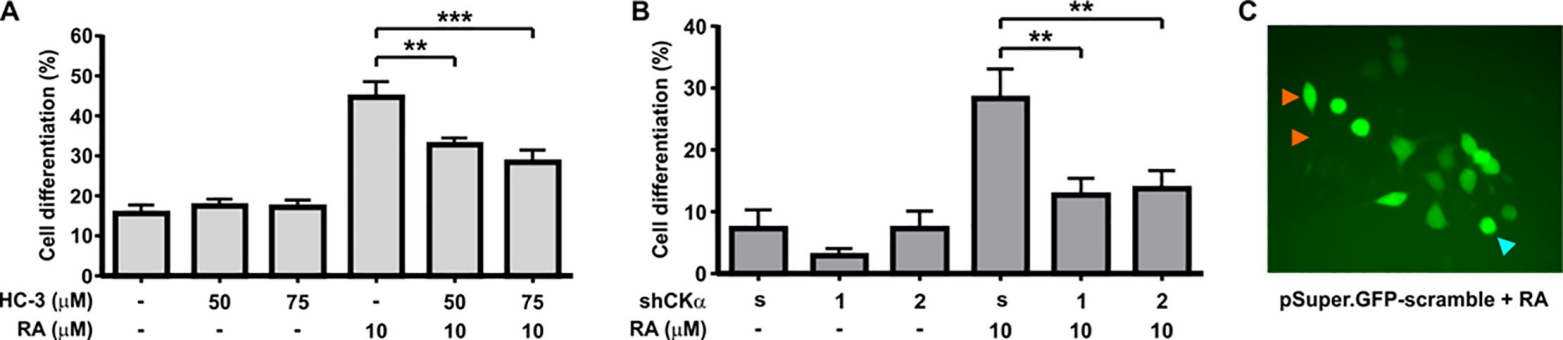
CKα activity and expression is essential for neuronal differentiation. **(A)** Neuro-2a cells were incubated under proliferation (-RA) or differentiation condition (+RA, 10μM), in the presence or absence of different concentration of Hemicholinium-3 (HC-3). Graph represents the percentage of neurite-bearing cells for each culture condition from four independent experiments. (B) Neuro-2a cells were transfected with the indicated plasmids designed to knocked down CKα expression, treated with or without RA (10 μM) and analyzed morphologically. (C) Representative image of transfected cell expressing pSuper.gfp/neo scramble. Orange head arrow indicates an example of a differentiated cell harboring a neurite, cyan head arrow shows an example of an undifferentiated neuron. *p<0.05, **p<0.001, ***p<0.0001.

CK0α plays a key role during neuronal differentiation [9]. In this context, CKα expression is tightly regulated; being low in undifferentiated neuroblasts and induced by RA coordinately with neuronal differentiation. In order to reinforce the role of CKα on neuronal differentiation, we analyzed the effect of its pharmacological inhibition and the knockdown of its expression using the shRNA strategy. First, we used hemicholinium-3 (HC-3) as a choline analog, to competitively inhibit CK activity [26,27]. Cells were incubated with 50 and 75 μM HC-3 under proliferation or differentiation conditions. In this last case, HC-3 was added 60 min before RA (10 μM). After 24 h of treatment, cells were morphometrically analyzed as previously described [28]. We observed that HC-3 has no effect on basal differentiation (proliferation condition) but clearly reduced differentiation induced by RA (Fig 1A). Then, with the propose of affecting CKα expression, we designed two plasmids to specifically knockdown CKα (see material and methods). The efficiency of each plasmid to block CKα expression was evaluated by western blot (S1 Fig). Each plasmid or the control (scramble) were transfected in Neuro-2a cells. After 48 h of transfection, RA was added and analyzed 24 h later. As transfected cells expressed GFP (pSuper.gfp/neo RNAi (OligoEngine)), the morphometric analysis was performed on green cells under fluorescence microscopy (Fig 1B and 1C). After RA induction, cells transfected with each shRNA show a clear reduction in the percentage of neuronal differentiation compared with cells transfected with the scramble. These results point out that a strict regulation of CKα expression is required for an adequate process of neuronal differentiation.

### KDM2B binds to the Box2 present in the *Chka-*proximal promoter

**Fig 2:**
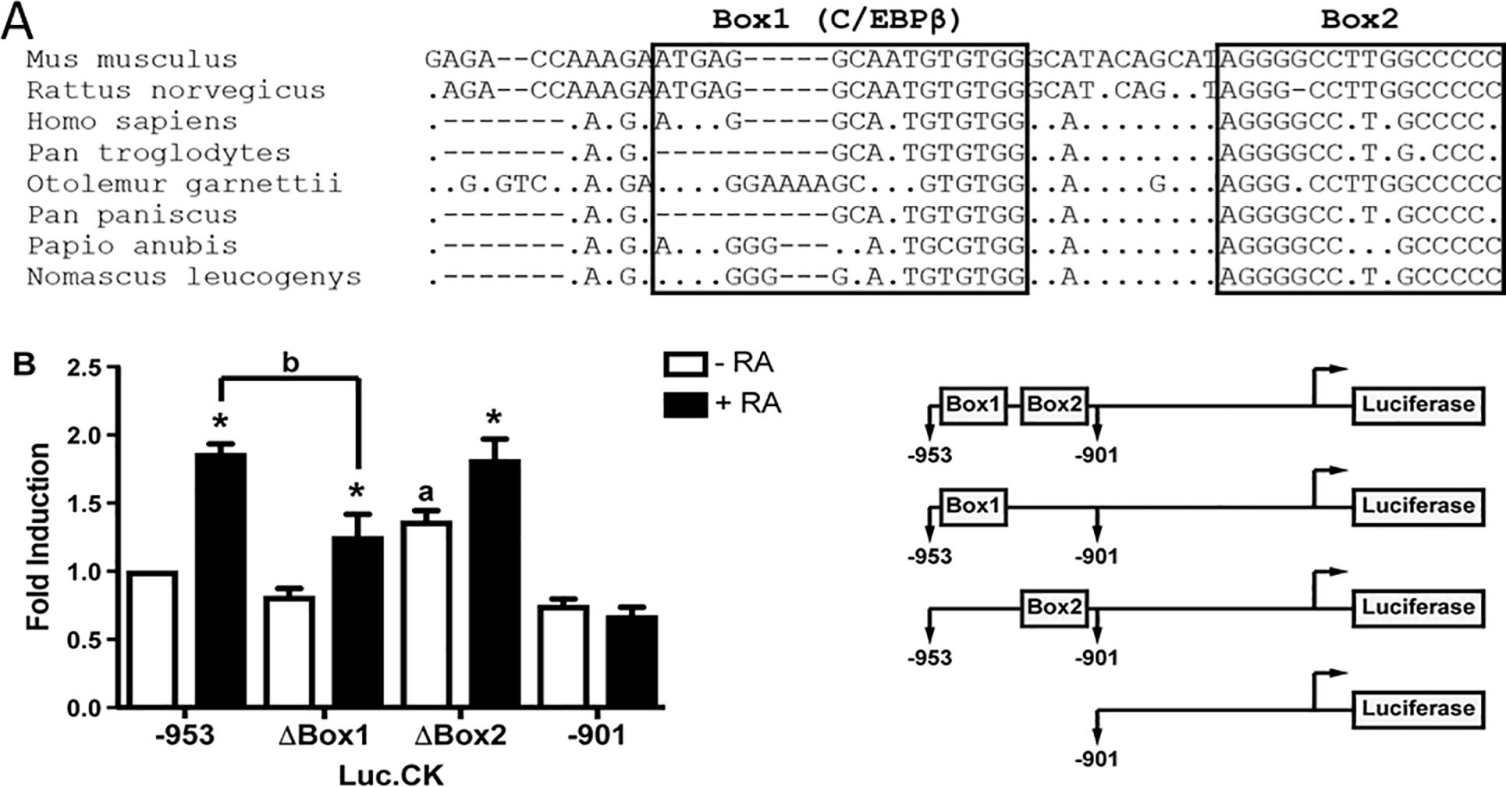
*Chka* proximal promoter region analysis. **(A)** Identification of conserved motives in the *Chka*-promoter (-953/-901 bp). Box1 and Box2 are highlighted. **(B)** Luciferase promoter assay. *Chka*-promoter reporter plasmids (0.5 μg) Luc.CK(-953/+57), Luc.CK(-901/+57), Luc.CKΔBox1, Luc.CKΔBox2 and pCMV-β-galactosidase plasmid (0.2 μg) were transfected in Neuro-2a cells. Luciferase activity is given relative to β-galactosidase activity and was measured 24 h after RA treatment. Graph represents the ratio Luciferase/ β-galactosidase ± S.D. obtained from four independent experiments. (*p<0.001, *a* indicates p<0.001 between Luc.CKΔBox2 and Luc.CK(-953/+57) basal activities, *b* indicates p<0.001 between Luc.CKΔBox2 and Luc.CK(-953/+57) activities in the presence of RA).

We have previously demonstrated that RA induces CKα transcription by a mechanism dependent on the levels of C/EBPβ and its binding to the element Box1 present in the *Chka-*proximal promoter. We also identified a highly conserved motive named Box2 [10] (Fig 2A). To farther characterize the regulation of CKα expression during neuroblasts differentiation, we analyzed the activity of promoter-reporter constructs harboring mutation in each of the identified elements, Box1 and/or Box2 (see schematic representation shown in Fig 2B). The analysis confirmed that the Box1 is involved in RA-induction [10]. In addition, deletion of the Box2 clearly alters the levels of CKα that characterized undifferentiated cells; in fact, deletion of Box2 induces the expression of CKα to similar levels to those reached during differentiation induced by RA treatment (Fig 2B). This result suggests that Box2 negatively regulates CKα expression under undifferentiated conditions.

### KDM2B binds to the Box2 present in the *Chka-*proximal promoter

**Fig 3:**
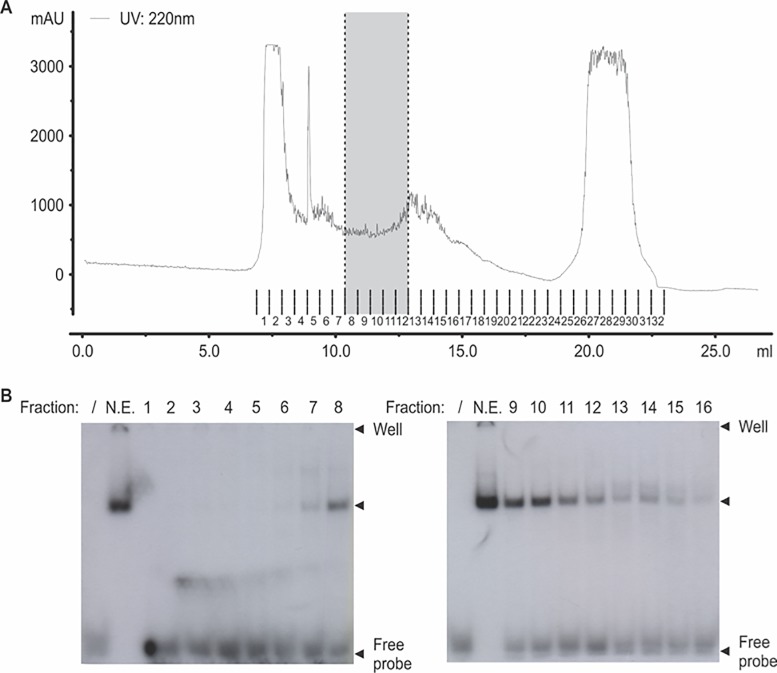
Box2 binding protein isolation and identification. **(A)** Picture of the elution profile of Neuro2-a nuclear proteins resolved by size exclusion chromatography using a Superdex S200 column. The region of the chromatogram comprising the fractions that retain the Box2 binding capacity are indicated in gray. **(B)** Representative image of EMSAs for different eluted fractions. Proteins from fractions from 8 to 12 (positive in the EMSA assay) were pooled together, selected by affinity chromatography and sequenced.

**Fig 4:**
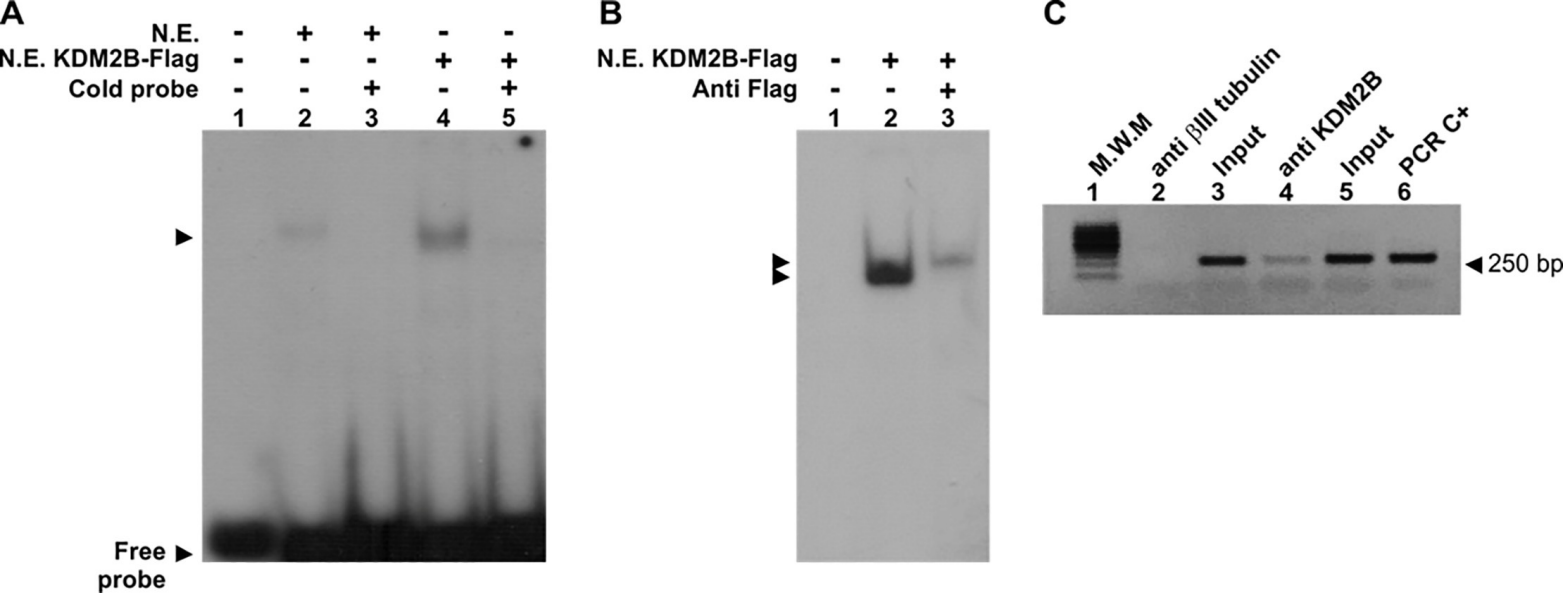
KDM2B bind *in vitro* and *in vivo* to Box2 present in *Chka* promoter. **(A)** EMSA assay. 25 bp [γ^32^P]-ATP labelled probe corresponding to the Box2 was incubated with nuclear extracts (NE) obtained from Neuro-2a cells or Neuro-2a cells transfected with a plasmid designed to overexpressed KDM2B-Flag (lanes 2 and 4 respectively) or without NE (lane 1). Arrows show the migration of the complexes. Lanes 3 and 5 show competition with excess of unlabeled probe (cold probe). (**B)** Super-shift assay. NE from KDM2B overexpressing Neuro-2a cells incubated with labeled Box2 probe and with (lane 3) or without (lane 2) anti-Flag antibody. (**C**) *In vivo* association between KDM2B and the CKα promoter. ChIP assay was performed with anti-KDM2B antibody. An unrelated antibody, anti-βIII-tubulin, was used as a control. Picture shows the electrophoresis gel by which the PCR products were resolved. Line 1: molecular weight marker, Line 2: anti-βIII tubulin antibody, Line 3: input, Line 4: anti-KDM2B antibody, line 5: input, Line 6 PCR positive control (mouse genomic DNA).

We have previously shown that a complex of proteins presents in nuclear extracts obtained from cells grown under proliferation and differentiation conditions binds to the element Box2 located in the *Chka* proximal promoter (EMSA assay). In addition, we have also demonstrated that the intensity of the complex increases when we assayed nuclear extracts that correspond to neuroblats (-RA) [10]. To identify the protein(s) that binds to the Box2, we purified this complex by size exclusion chromatography followed by an affinity column (see material and methods) (Fig 3A and 3B). The fractions containing the proteins eluted from the labeled probe were analyzed by nLC-ESI-MS/MS spectrometer (Structural Biology and Proteomic Service, Autonomous University of Barcelona). From the identified proteins by mass spectrometry (Table 2), the histone lysine demethylase KDM2B is the only protein able to bind to the DNA, consequently, we evaluated its affinity to the Box2 by *in vitro* EMSA assay. For this porpuse, cells were transfected with a plasmid designed to overexpressed KDM2B-tagged to Flag (gently provided by Dr. Klose [23]) or the empty plasmid as a control, and nuclear extracts were prepared from cells grown under proliferation condition (-RA). The EMSA assay was performed using labeled-Box2 as a probe. As Fig 4A shows, we were able to detect a complex when we used nuclear extract obtained from cells transfected with KDM2B-Flag (line 4), and the intensity of this complex overcome to that obtained from cells transfected with the empty plasmid (line 2). A competition with cold probe disassembles the complex confirming the specificity of the binding (lines 3 and 5). When we added anti-Flag antibody after incubation of the extracts with the probe, a super-shift was observed, suggesting that the specific antibody binds to the complex (Fig 4B).

**Table 2:** Proteins identified by mass spectrometry.

	Name	Domain	Function
Casd1	CAS1 domain containing 1	Acyl transferase	Modify cell-surface biopolymers such as glycans and glycoproteins.
		PC-esterase	
Slc7a2	Solute carrier family 7, member 2	AA permease C	Cationic amino acid transporter
		Low affinity cationic amino acid transporter 2	
		SLC5-6-like sbd	
Pdia3	Protein disulfide isomerase associated 3	Thioredoxin	poly(A) RNA binding
		Protein disulfide-isomerase	protein disulfide isomerase activity
Map2k1	Mitogen-activated protein kinase kinase 1	Serine/Threonine protein kinases	Cell signalling
Thbs4	Thrombospondin 4	Cartilage oligomeric matrix protein	Calcium, extracellular matrix and protein binding
		Thrombospondin N-terminal -like domains	
		Calcium-binding EGF-like domain	
Gucy1a3	Guanylate cyclase 1, soluble, alpha 3	Adenylyl- / guanylyl cyclase, catalytic domain	Cell signalling
		Heme NO binding associated	
Wdr24	WD repeat domain 24	WD40 domain	???
Polh	DNA polymerase eta	Nucleotidyltransferase/DNA polymerase	DNA synthesis
Srpr	Signal recognition particle receptor	Signal recognition particle	Docking protein
Ints3	Integrator complex subunit 3	single-stranded DNA binding	DNA repair
Nod2	Nucleotide-binding oligomerization domain containing 2	Leucine-rich repeats	Bacterial sensor, immune response
		Death Domain	
Ush2a	Usher syndrome 2A	Protein binding domain	Protein interaction
Kdm2b	Lysine (K)-specific demethylase 2B	N-terminal jumonji C domain	H3K36-specific histone demethylase
		CxxC zinc finger domain	leukemia maintenance and development
		plant homeodomain finger	maintenance of mouse embryonic stem cell pluripotency
		F-box	induced pluripotent stem cell generation
		leucine-rich repeats	negative regulation transcription RNA polimerase II promoter
Tatdn2	TatD DNase domain containing 2	magnesium dependent DNase activity	DNase
Atp1a1	ATPase, Na+/K+ transporting, alpha 1 polypeptide	Cation transporting ATPase	Inorganic cationic transporter

Reinforcing these results, we also demonstrated that KDM2B binds *in vivo* to the Box2 by performing ChIP analysis. As Fig 4C shows, KDM2B specific antibody but not the unrelated βIII-tubulin antibody was effective in pulled down the *Chka* promoter, specifically amplified by PCR reaction (compare lines 2 and 4). In conclusion, under proliferating condition, KDM2B binds to the Box2 present in the *Chka* promoter.

### CKα and KDM2B expression during RA-induced neuronal differentiation

**Fig 5:**
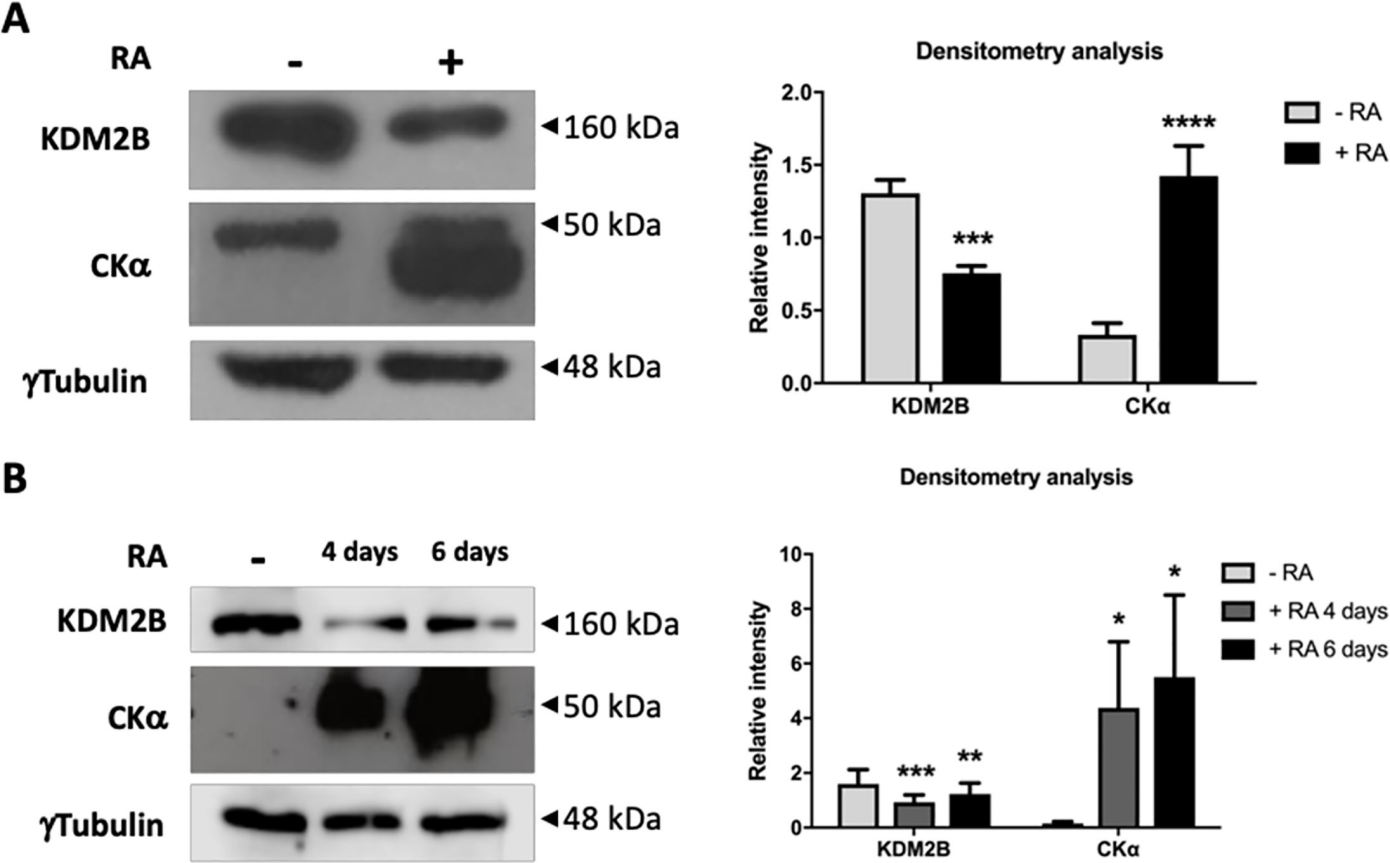
KDM2B and CKα expression during neuronal differentiation. Total cellular extracts obtained under proliferation (-RA) or differentiation condition (+ RA) were analyzed by western blot to detect the pattern of KDM2B and CKα expression. γ-Tubulin was used a loading control. (**A)** Neuro-2a cells were treated with RA during 48 h. (**B)** SH-SY5Y cells were treated with RA during 4 and 6 days. *p<0.05, **p<0.01, ***p<0.001, ****p<0.0001.

We evaluated the expression of KDM2B and CKα during RA-induced neuronal differentiation of mouse Neuro-2a and human SH-SY5Y cells by western blot. As expected, CKα is induced by RA [9,10], while KDM2B shows an opposite pattern of expression, with higher levels in the control, corresponding to undifferentiated condition, and a decreased with RA-induced differentiation (Fig 5A and 5B). This expression pattern fits with previous results showing that there is more binding to the Box2 when we assayed by EMSA nuclear extracts obtained from undifferentiated cells, and also with a possible role of KDM2B as a negative regulator of CKα expression through the binding to the Box2 (Fig 2B) [10].

### KDM2B knockdown affects CKα expression an cell differentiation

**Fig 6:**
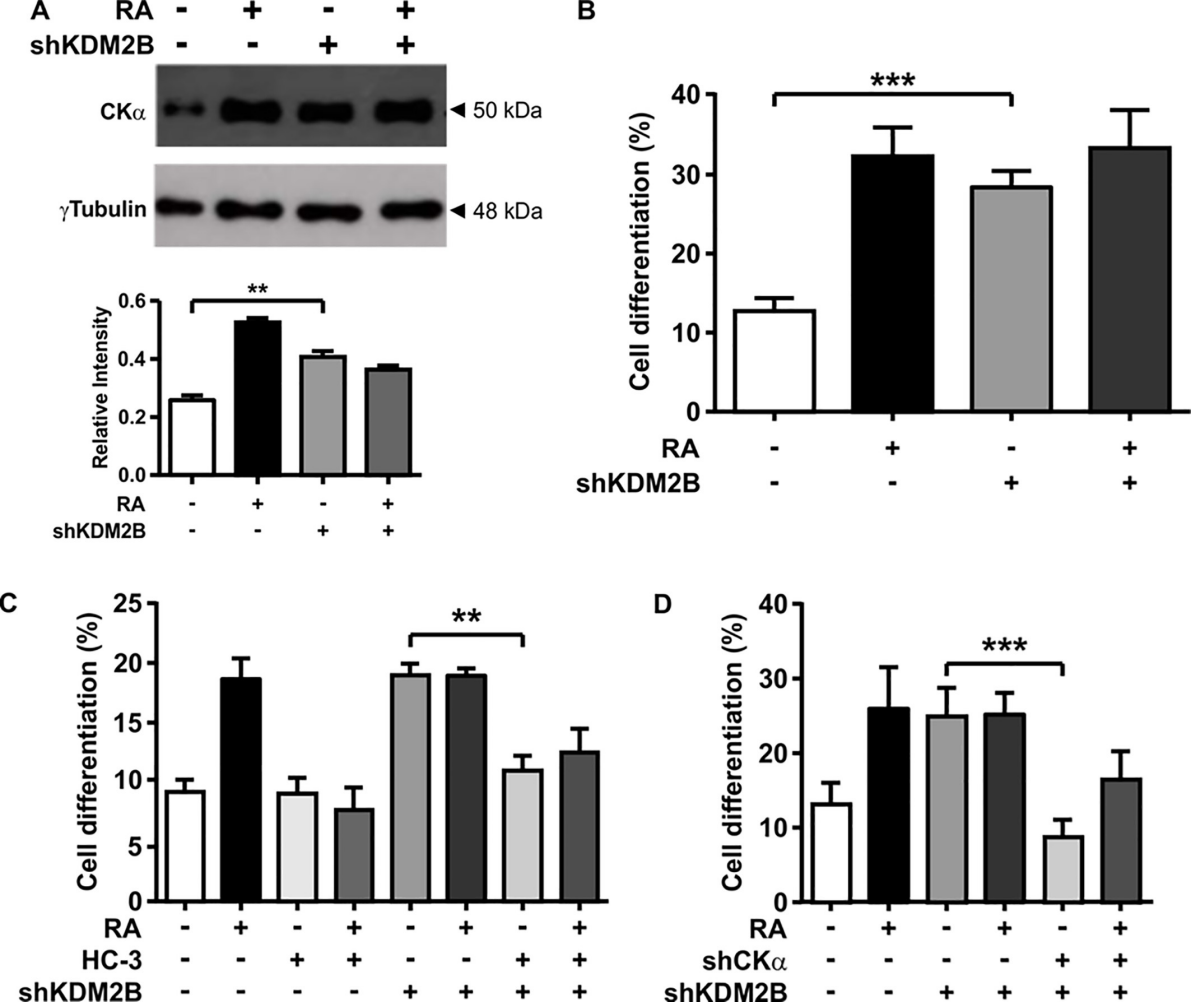
KDM2B knockdown induces CKα expression and neuronal differentiation. **(A)** Western blot analysis of CKα expression obtained from total cellular extract of control cells or cells transfected with shKDM2B. γTubulin was used as loading control. (**B**) Neuro-2a cells were transfected with the indicated plasmids designed to knocked down KDM2B expression, treated with or without RA (10 μM) and analyzed morphologically. **(C)** Neuro-2a cells were transfected with the indicated plasmids designed to knocked down CKα and or KDM2B expression, treated with or without RA (10 μM) and analyzed morphologically. (**D**) Neuro-2a cells were transfected with the indicated plasmids designed to knocked down KDM2B expression, treated with or without HC-3 and analyzed morphologically. *p<0.05, **p<0.001, ***p<0.0001.

To further elucidate the role of KDM2B as a regulator of *Chka* gene transcription, we monitored by western blot the expression of CKα in cells grown under differentiation or proliferation conditions when KDM2B expression was downregulated by siRNA technology. The efficiency of the knockdown was demonstrated by co-transfection of KDM2B-Flag together with sh-KDM2Bs or the sh-scramble and by assaying with anti-Flag antibody. We only detected a 160kDa band corresponding to KDM2B-Flag when we co-transfected with sh-scramble (S2 Fig). In order to avoid cell adaptation, the analysis was performed with transient transfection assays. When we analyzed CKα expression under condition of KDM2B knockdown (cells transfected with sh1-KDM2B plus sh2-KDM2B), an altered pattern of expression was detected. In fact, Neuro-2a control cells (sh-scramble) showed the previously described increased in CKα expression with neuronal differentiation (+RA). However, this profile was lost in cells treated with sh-KDM2B which showed similar levels of CKα in both conditions (-RA or +RA), and higher compared with the control (-RA) (Fig 6A). Interestingly, the lack of difference is due to an increased in the basal levels of CKαexpression in undifferentiated cells rather than a defect in RA-induction. Similar results were observed in luciferase reporter assays (see Fig 2B). These results suggest that KDM2B acts as a negative regulator of CKα expression in neuroblast cells (-RA).

### KDM2B regulates neuronal differentiation by altering CKα expression

To evaluate neuronal differentiation in conditions where KDM2B expression is downregulated, we performed morphometric analysis of neuroblast cells showing green fluorescence (material and methods). As Fig 6B shows, downregulation of KDM2B promotes neuronal differentiation even without RA treatment (MEM 10% FBS).

We have previously demonstrated that CKα overexpression in Neuro-2a cells provokes cell differentiation even in the absence of RA [9]. As KDM2B knockdown induces cell differentiation of neuroblast cells (Fig 6B), we asked whether or not these effect is dependent on the induction of CKα expression. Alternatively, KDM2B could regulate neuronal differentiation by an independent mechanism as was described for adipocyte differentiation [17]. To evaluate the first possibility, neuronal differentiation was analyzed when both KDM2B and CKα were inhibited. For this, we transfected neuroblast cells with the designed shRNA to knockdown KDM2B and cells were treated with Hemicholineum-3 as a CKα inhibitor. Cells differentiation of GFP-expressing cells was evaluated after 24h. In a second experiment, we knockdown CKα expression using two specific shRNA (sh1-CKα and sh2-CKα) (Fig 1B); for this experiment we co-transfected cells with sh-KDM2B and sh-CKα or the sh-scramble. As Fig 6C and 6D show, when CKα was inhibited or downregulated, cell differentiation promoted by KDM2B downregulation was blocked in both cases. Thus, the effect of KDM2B on cell differentiation of neuroblast cells is dependent on CKα overexpression.

## Discussion

During RA-induced differentiation of neuroblastoma cells, the demand for membrane biosynthesis that accompanies neuritogenesis is covered by an increase in PtdCho biosynthesis. PtdCho biosynthesis rises by two mechanisms: the early one involves the enzymatic activation of CDP–choline: 1,2-diacylglycerol cholinephosphotransferase (CPT) and CCT, and the second and late mechanism involves the transcriptional activation of CKα and CCTα expression [9].

We now demonstrate for the first time, that CKα is essential for Neuro-2a cells differentiation. By using a pharmacological inhibitor of CKα or by affecting its expression using two different siRNAs, we showed that the lack of CKα expression or activity impairs neuronal differentiation (Fig 1). These results clearly show that CKα plays a key role in supporting PtdCho biosynthesis required for neuronal differentiation.

We have previously demonstrated that after RA treatment, CKα expression is transcriptionally induced by the binding of C/EBPβ to the Box1 located (−953/−901) upstream of the start transcriptional point of the *Chka* gene [10]. This region also contains an inverted repeat sequences named Box2, which is involved and required to reach the full transcription of *Chka* [10]. The present work provides mechanistic details about the transcriptional regulation of *Chka* in neuroblasts cells. A transcription factor search did not reveal any consensus binding sites in the element Box2, consequently, we purified the protein complex by size exclusion chromatography, followed by the affinity column. The nLC-ESI-MS/MS spectrometer analysis revealed the presence of the protein KDM2B (Fig 3). In order to confirmed its participation in *Chka* regulation, we demonstrated by *in vivo* and *in vitro* assays that KDM2B binds to the Box2 in the *Chka* promoter (Fig 4).

The functional role of KDM2B was evaluated by promoter reporter assays (Fig 2) and by affecting KDM2B expression using two different shRNA expressing plasmids (Fig 6). The results obtained with both experiments demonstrated that KDM2B negatively regulate CKα expression to ensure the low levels characteristic of proliferative neuroblast cells; either deletion of Box2 or inhibition of KDM2B expression induces CKα expression in proliferative neuroblasts.

We have previously showed that CKα overexpression drives neuroblast cells to neuronal differentiation even in the absence of RA [9]. As KDM2B knockdown provokes a clear induction in CKα expression, we quantified neuronal differentiation by morphometric analysis and demonstrated that under this conditions neuroblast cells undergo differentiation even in the absence of RA (Fig 6).

It was shown that KDM2B is an anti-adipogenic factor that is up-regulated during the early phase of 3T3-L1 preadipocyte differentiation [17]. To evaluate if the effect of KDM2B on neurogenesis depends on CKα induction, we evaluated differentiation in cells with deficiency of both KDM2B and CKα. As inhibition of CKα activity or expression restored the levels of cell differentiation, we confirmed that KDM2B indirectly regulated cell differentiation by altering CKα expression (Fig 6).

**Fig 7:**
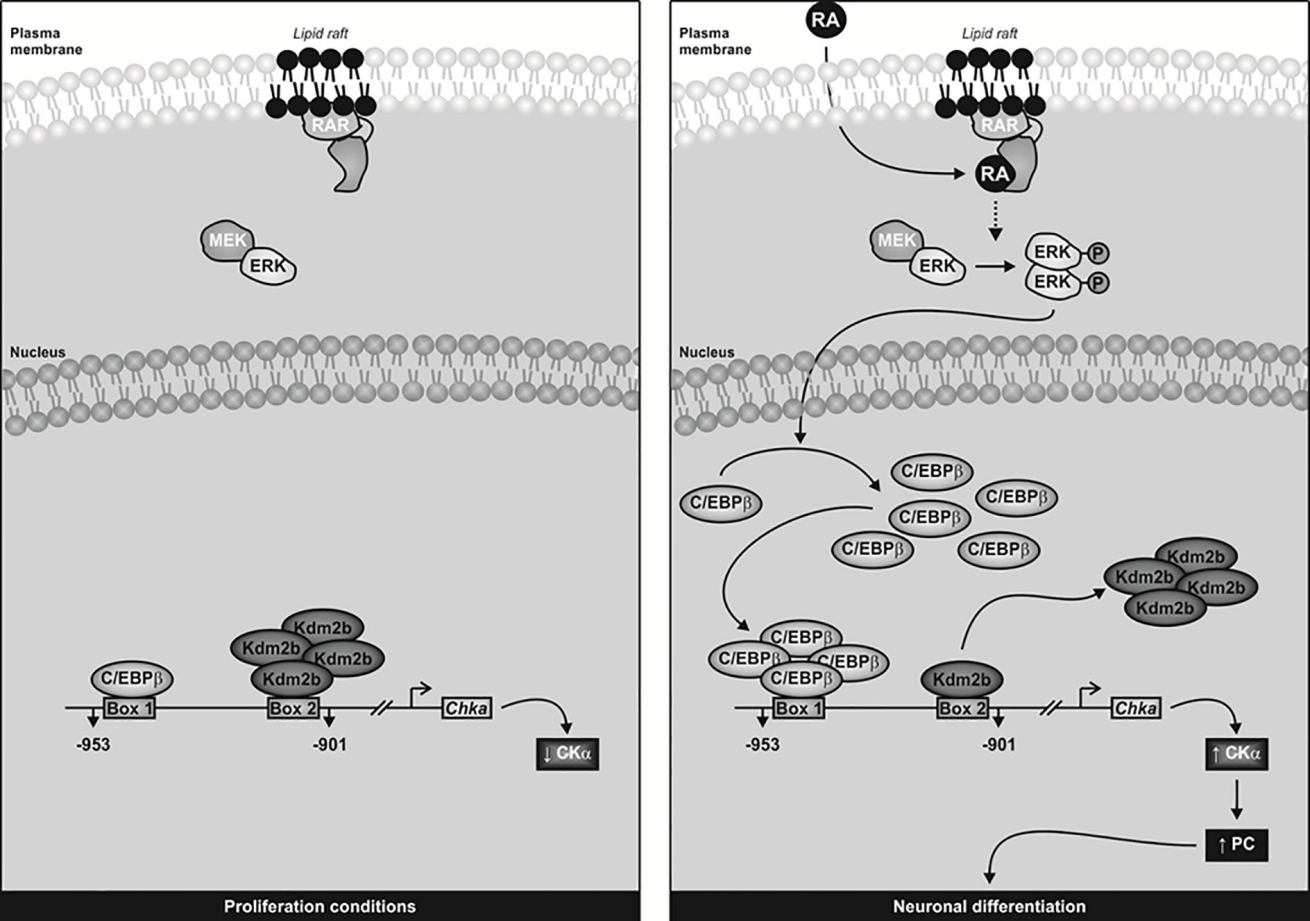
Shematic representation of *Chka* gene-expression during neuronal differentiation. Under proliferation conditions, the expression of CKα is low due to the binding of KDM2B to the Box2 in the *Chka*-proximal promoter and the repression of its transcription. High levels of KDM2B expression are charactheristic of neuroblastoma cells. After RA-induced neuronal differentiation, ERK pathways is activate and induces both, the expression of C/EBPβ and its binding to the Box1 and a decrease in KDM2B expression. These events induces CKα expression and, as a consequence, neuronal differentiation.

In conclusion, we contribute to the understanding of the model of CKα regulation during neuronal differentiation which is schematically represented in Fig 7. During proliferation in the absence of RA, ERK is inactive, and low levels of C/EBPβ occupies the Box1 [10]. Moreover, there is an induction of KDM2B expression. The binding of KBM2B to the Box2 probably as a complex with other not yet identified proteins represses CKα expression and maintains the undifferentiated and proliferative state of this tumor cells. We cannot discard the possibility that KDM2B blocks ERK activation as was described in others model of undifferentiated cells like cancer cells [29]. After RA induction, the ERK-dependent induction of C/EBPβ expression and its binding to the Box1, promote the transcriptional induction of CKα. Under this condition, we detected low levels of KDM2B expression and weak binding to the Box2.

In addition, our finding provides novel data about the role of KDM2B in maintaining the undifferentiated and proliferative state of neuroblast cells. In fact, we have demonstrated in cells model that the level of KDM2B expression are higher in neuroblast cells (Fig 5). This is also true for samples from neuroblastoma patients which show an association between levels of KDM2B and poor prognostic (S3 Fig). We also shown that even in the absence of RA, neuronal differentiation enhanced by bloking the expression of KDM2B (Fig 6). These results could explain recent work demonstrating that inhibition of histone demethylaion by GSK-J4 is effective in high-risk neuroblastoma by inducing cell differentiation [21]. All together we would like to propose that the levels of KDM2B could be an important tool for diagnostic, treatment and prognosis of this lethal cancer.

## Supporting information

S1 Fig sh-CKα efficiency. (TIF)

S2 Fig sh-KDM2B efficiency. (TIF)

S3 Fig Higher expression of KDM2B are related with poor prognosis of neuroblastoma. (TIF)
