# Supplementary figures and images for: KDM2B regulates choline kinase expression and neuronal differentiation of neuroblastoma cells
Source: PLoS One. 2019 Jan 10;14(1):e0210207. doi: 10.1371/journal.pone.0210207 (PMC6328129; doi:10.1371/journal.pone.0210207)

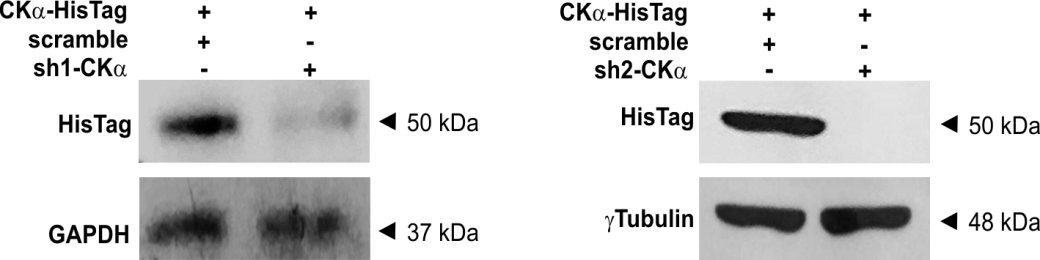

Supplement: S1 Fig — (TIF) [file pone.0210207.s001.tif]

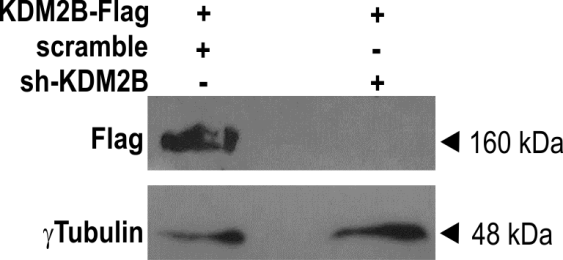

Supplement: S2 Fig — (TIF) [file pone.0210207.s002.tif]

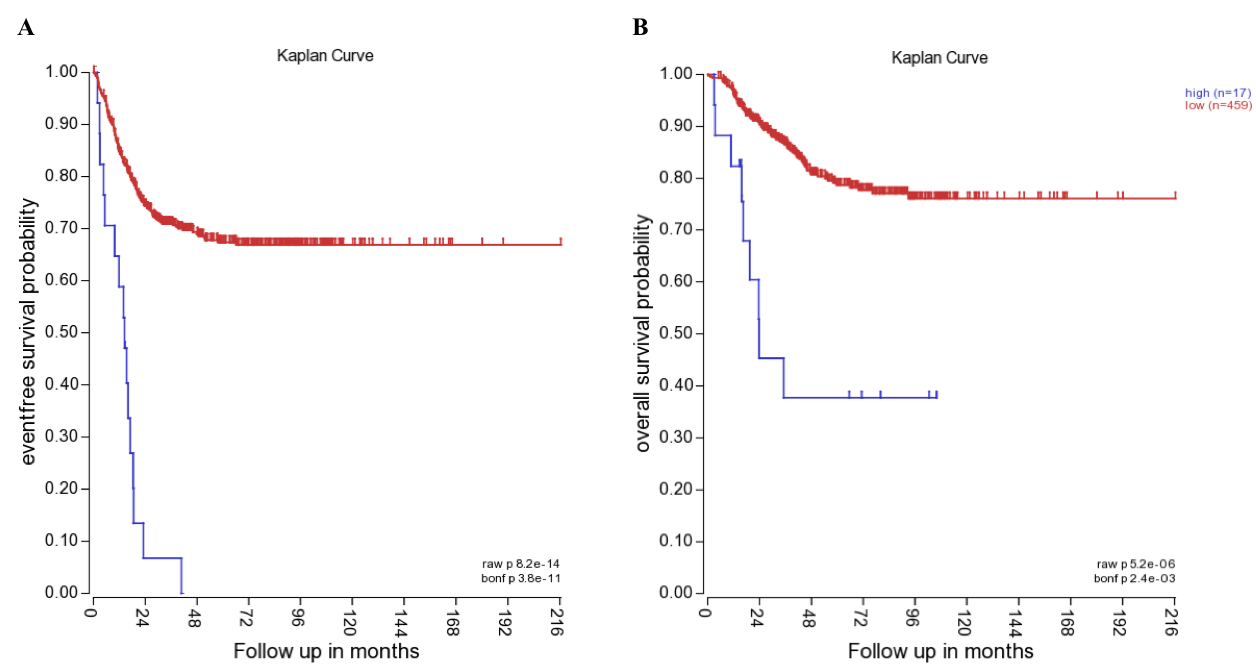

Supplement: S3 Fig — (TIF) [file pone.0210207.s003.tif]
